# Biological Potential of *Asphodelus microcarpus* Extracts: α-Glucosidase and Antibiofilm Activities In Vitro

**DOI:** 10.3390/molecules29215063

**Published:** 2024-10-26

**Authors:** Sonia Floris, Francesca Pintus, Antonella Fais, Benedetta Era, Nicola Raho, Chiara Siguri, Germano Orrù, Sara Fais, Carlo Ignazio Giovanni Tuberoso, Stefania Olla, Amalia Di Petrillo

**Affiliations:** 1 Department of Life and Environmental Sciences, University of Cagliari, SS 554-Bivio per Sestu, Cittadella Universitaria, 09042 Monserrato, Italy; s.floris@unica.it (S.F.); fpintus@unica.it (F.P.); era@unica.it (B.E.); tuberoso@unica.it (C.I.G.T.); 2 Gastroenterology Unit, Department of Medical Science and Public Health, University of Cagliari, Cittadella Universitaria, 09042 Monserrato, Italy; nicola.raho@unica.it (N.R.); amalia.dip@unica.it (A.D.P.); 3 Institute for Genetic and Biomedical Research (IRGB), The National Research Council (CNR), 09042 Monserrato, Italy; chiara.siguri@irgb.cnr.it; 4 Department of Surgical Sciences, University of Cagliari, Cittadella Universitaria, 09042 Monserrato, Italy; orru@unica.it (G.O.); sarafais79@gmail.com (S.F.)

**Keywords:** *Asphodelus microcarpus*, α-glucosidase, α-amylase, molecular docking

## Abstract

Type 2 diabetes (T2D), characterized by insulin resistance and β-cell dysfunction, requires continuous advancements in management strategies, particularly in controlling postprandial hyperglycemia to prevent complications. Current antidiabetics, which have α-amylase and α-glucosidase inhibitory activities, have side effects, prompting the search for better alternatives. In addition, diabetes patients are particularly vulnerable to yeast infections because an unusual sugar concentration promotes the growth of *Candida* spp. in areas like the mouth and genitalia. *Asphodelus microcarpus* contains bioactive flavonoids with potential enzyme inhibitory properties. This study investigates α-amylase and α-glucosidase inhibitory activities and antioxidant and antimycotic capacity of ethanolic extracts from different parts of *A. microcarpus*. Results show that extracts significantly inhibit α-glucosidase, with the IC_50_ value being up to 25 times higher than for acarbose, while exerting low α-amylase activity. The extracts also demonstrated strong antioxidant properties and low cytotoxicity. The presence of phenolic compounds is likely responsible for the observed biological activities. Molecular docking analysis of 11 selected compounds identified emodin and luteolin as significant inhibitors of α-glucosidase. Additionally, the extracts demonstrated significant antibiofilm action against an MDR strain of *Candida albicans*. These findings suggest that *A. microcarpus* is a promising source of natural compounds for T2D management.

## 1. Introduction

Diabetes, a significant global health concern, impacts millions of individuals worldwide, underscoring the need for continuous advancements in effective management strategies [1]. While type 1 diabetes primarily necessitates insulin therapy due to pancreatic β-cell dysfunction, type 2 diabetes (T2D) is characterized by insulin resistance and a progressive decline in β-cell function [2]. Managing T2D requires comprehensive strategies to control blood glucose levels and minimize the risk of complications. A critical component of T2D management is the regulation of postprandial hyperglycemia, which is essential in averting long-term vascular complications [3]. Currently, several antidiabetic drugs, such as acarbose, inhibit α-amylase (EC 3.2.1.1) and α-glucosidase (EC 3.2.20). α-Amylase hydrolyzes starch into smaller molecules, while α-glucosidase is an exoenzyme that cleaves disaccharides and oligosaccharides into glucose in the small intestine [4]. Excessive inhibition of α-amylase is associated with side effects, such as gastrointestinal discomfort [5], highlighting the need for more tolerable and efficacious alternatives.

Beyond glucose management, diabetes patients are also predisposed to infections due to compromised immune function. Among the most common are fungal infections, particularly those caused by *Candida albicans*. Individuals with diabetes, especially those with poorly controlled blood glucose levels, are at a higher risk of candidiasis, as hyperglycemia fosters an environment conducive to fungal overgrowth [6]. *C. albicans* is an opportunistic pathogen that causes infections ranging from superficial mucosal involvement to invasive, life-threatening infections in diabetic patients. Managing such infections is crucial to prevent further complications, as fungal overgrowth can exacerbate inflammation and damage in vulnerable tissues [7].

Research has shown that several plants contain substances that can inhibit the activity of α-amylase and α-glucosidase enzymes [8,9]. Flavonoids are revealed to be of particular interest in the inhibition of these enzymes [10,11]. Genus *Asphodelus* spp., renowned for its historical use in folk medicine, has emerged as a novel research domain, courtesy of its prospective bioactive properties, such as antimicrobial and antimycotic activity [12]. *A. microcarpus*, a significant plant in the Liliaceae family, is utilized in traditional medicine for treating local inflammation, with its roots employed against white spots and specifically for ear pain. Various studies have validated the antioxidant, antimelanogenic, anti-inflammatory, and antimicrobial activities of *A. microcarpus*, corroborating its ethnopharmacological applications and providing a scientific basis for its usage in folk medicine [13,14]. Furthermore, *A. microcarpus* is known for its rich phenolic content, and its flowers are laden with antioxidant molecules like luteolin and its derivatives. A high concentration of flavonoids in aerial parts of *A. microcarpus* was also detected in our previous study [13,15]. In particular, the extracts presented luteolin and its glycosylated derivates in high concentrations. These extracts also showed good antioxidant activities. As reported by Kim and coworkers [16] luteolin and some glycosylated derivates showed potent inhibitory activity against α-amylase and α-glucosidase.

Considering these findings, our study aims to investigate the potential of *A. microcarpus* as a valuable source of natural compounds capable of efficiently inhibiting enzymes like α-amylase and α-glucosidase. This exploration is intended to unlock the therapeutic potential of *A. microcarpus* in the context of natural medicine, further contributing to our understanding of its health-promoting properties, particularly regarding diabetes management and infection control.

## 2. Results

### 2.1. Enzymatic Inhibition

The prepared extracts, as described in the Materials and Methods, were evaluated for their inhibitory activity against α-glucosidase and α-amylase. The extracts were tested at a concentration of 100 µg/mL and the results are presented in Table 1.

Our results demonstrated that the EE of *A. microcarpus* completely inhibited α-glucosidase enzyme at the dose tested, while showing relatively low activity towards the α-amylase enzyme.

Based on these results, we calculated the IC_50_ values only for the α-glucosidase enzyme. As shown in Table 2, all extracts exhibited an IC_50_ value statistically lower than that of acarbose (*p* < 0.0001). The extracts were up to 25 times more effective than the standard inhibitor.

These preliminary results indicate that the extracts of *A. microcarpus* show interesting potential as inhibitors of the α-glucosidase enzyme.

### 2.2. Antimycotic Activity

The results obtained using the microplate procedure displayed an antibiofilm activity for some ethanol extracts. Furthermore, none of the formulations inhibited the planktonic form of Candida albicans at the tested concentrations (MIC > 4 mg/mL). According to Table 3, ethanol base formulations made from stems, leaves, and flowers exhibited the lowest minimum biofilm inhibitory concentration (MBIC) values when compared to ethanol-extracted tubers; the first cited formulations showed an MBIC equal to 1.3 mg/mL.

### 2.3. Cell Viability and Cellular Antioxidant Activity

As previously reported, the EEs of *A. microcarpus* exhibited good antioxidant activity [13] on ABTS and DPPH assay. Therefore, we investigated their potential to inhibit H_2_O_2_-induced ROS generation in a cellular model.

**Figure 1:**
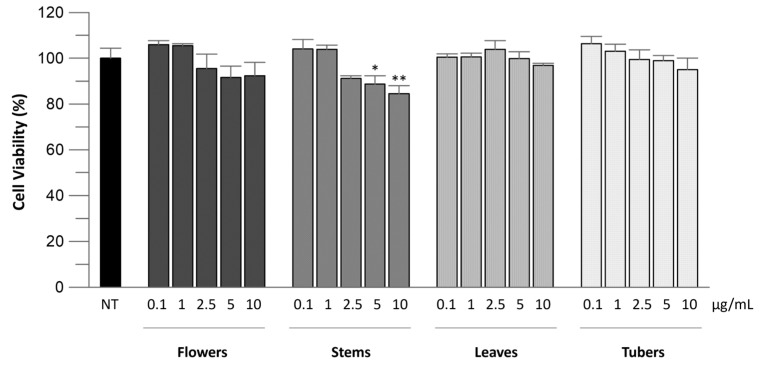
Effect of EEs from flowers, stems, leaves, and tubers of *A. microcarpus* on Caco-2 cell viability. After 24 h of incubation with samples at different concentrations (0.1–10 μg/mL), cell viability was evaluated by the MTT assay. Asterisks indicate statistically significant differences between non-treated (NT) cells and stem samples at concentrations of 5 μg/mL (* *p* < 0.01) and 10 μg/mL (** *p* < 0.0001).

First, we checked if the extracts could affect cell viability, using Caco-2 cells as a cellular model. EEs from flowers, stems, leaves, and tubers were tested at concentrations from 0.1 to 10 µg/mL. The results showed that none of the extracts was cytotoxic since the cell viability remained over 84% in all samples (Figure 1). In particular, all the extracts had no effect at 0.1 and 1 µg/mL, with leaves and tubers exerting no effect up to the higher concentrations tested. Only a slight decrease was observed for flower and stem extracts at concentrations of 2.5, 5, and 10 µg/mL with percentages of viability above 91% for flowers and from 91.2 to 84.4% for stems. These values are, however, not statistically significant, except for the stems at the concentrations of 5 (*p* < 0.01) and 10 µg/mL (*p* < 0.0001).

**Figure 2:**
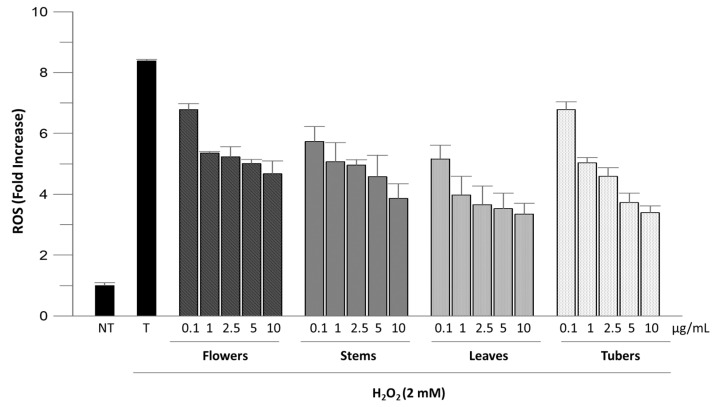
Inhibition of H_2_O_2_-induced ROS generation by ethanolic extracts of *A. microcarpus* on Caco-2 cells. NT, non-treated cells; T, cells treated with H_2_O_2_ only. All H_2_O_2_-treated samples were statistically different from cells treated with H_2_O_2_ only (*p* < 0.0001).

In the cellular antioxidant assay, ROS levels in Caco-2 cells were evaluated before and after H_2_O_2_-induced oxidative stress and upon treatment with extracts at different concentrations (0.1–10 µg/mL). Figure 2 shows that the treatment with only H_2_O_2_ significantly increased ROS levels (T vs. NT cells; *p* < 0.0001). All tested extracts demonstrated a significant cellular antioxidant activity reducing fluorescence emission in dose-dependent manner. The reduction in fluorescence emission indicates a decrease in ROS levels on cells treated with the extracts. This effect can be attributed to the action of phenolic compounds present in the extracts, which have been shown to possess antioxidant capabilities. All samples reduced fluorescence emission by about 50% at a concentration of 10 µg/mL. Considering each concentration, the greatest intracellular ROS inhibition was provided by leaf extract (Figure 2).

### 2.4. (HR) HPLC-ESI-QToF MS and (HR) HPLC-ESI-QToF MS/MS Analysis of A. microcarpus Extracts

**Figure 3:**
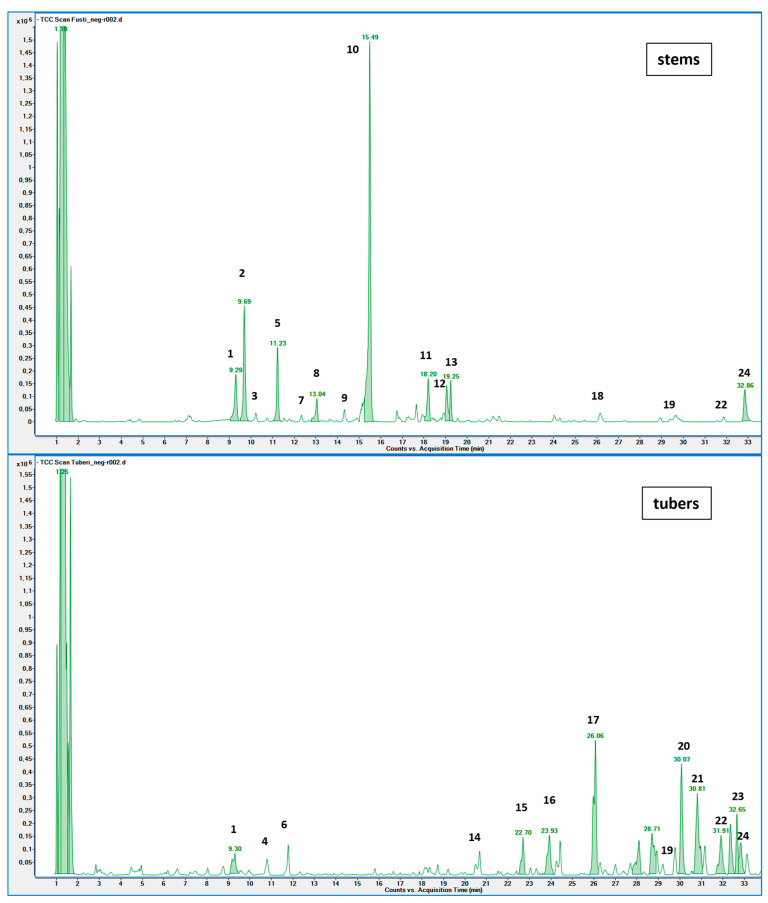
LC-ESI-Orbitrap-MS chromatograms of extracts obtained from the stems and tubers of *A. microcarpus*.

The EEs obtained from *A. microcarpus* leaves, stems, flowers, and tubers were qualitatively analyzed by (HR) HPLC-ESI-QToF MS/MS in negative ion mode, and the profile showed the presence of various compounds (Table 4), mainly belonging to the classes of polyphenols, anthrones/anthraquinones, and furanocoumarins. Figure 3 reports the LC-ESI-Orbitrap-MS of *A. microcarpus* stem (SEE) and tuber ethanol extract (TEE) (chromatograms of FEE and LEE extracts were previously published [13,15]). Several phenolic compounds, namely 3-O-caffeoylquinic acid (2), 5-O-caffeoylquinic acid (5), luteolin 6-C-glucoside (10), luteolin O-acetylglucoside (13), and luteolin (18), were previously detected in *A. microcarpus* FEE and LEE [13,15]. Compound 7 with molecular ion at *m*/*z* [M−H]^-^ 337.0938, corresponding to a molecular formula of C_16_H_18_O_8_ and main fragment ion at *m*/*z* 191.0560 was attributed to coumaroyl quinic acid [17]. Luteolin-6-C-glucoside was the most abundant peak in *A. microcarpus* SEE, LEE, and FEE, while no traces were found in TEE. Also, aloesin (1), a C-glycosylated chromone previously detected in *A. microcarpus* TEE [18], was detected for the first time in the other studied parts of *A. microcarpus*. Several anthraquinones were found in TEE, as well as emodin (4), aloinoside A/B (16), and laccaic acid (19). Emodin was already detected in *A. microcarpus* tubers [19], while aloinoside A/B and laccaic acid were found in Aloe species [20] and Asphodelus species [21], respectively. Compound 15 showed a molecular ion at *m*/*z* [M−H]^-^ 577.1577, corresponding to a molecular formula of C_27_H_30_O_14_ and main fragment ion at *m*/*z* 253.0507. Taking into account the presence of chrysophanol in *A. microcarpus* tubers [19], the compound was tentatively identified as a chrysophanol diglucoside [22]. Other anthrones and anthraquinones were reported in *A. microcarpus* tubers [18,19], but they were not found in the extracts obtained in this experimentation.

### 2.5. Molecular Docking

To identify the compounds in EE responsible for inhibiting α-glucosidase, we docked 11 compounds. Our selection of these 11 compounds was strategic. We included aloesin, a compound consistently found in all four ethanol extracts (SEE, LEE, TEE, and FEE). Additionally, we incorporated four compounds that were present in extract T and six compounds that were common across extracts S, L, and T. This comprehensive selection aimed to cover the most likely candidates responsible for the observed inhibitory activity.

Table 5 shows the structures and results of all 11 selected compounds docked in the α-glucosidase enzyme. The results indicate that the most promising compounds exhibiting the best binding affinity for α-glucosidase are emodin (−6.14 kcal/mol) and luteolin (−6.65 kcal/mol), whereas all others display energies inconsistent with enzyme binding affinity, suggesting a lack of activity. Our findings are supported by literature data, which report the in vitro activity of both emodin and luteolin [23,24].

**Figure 4:**
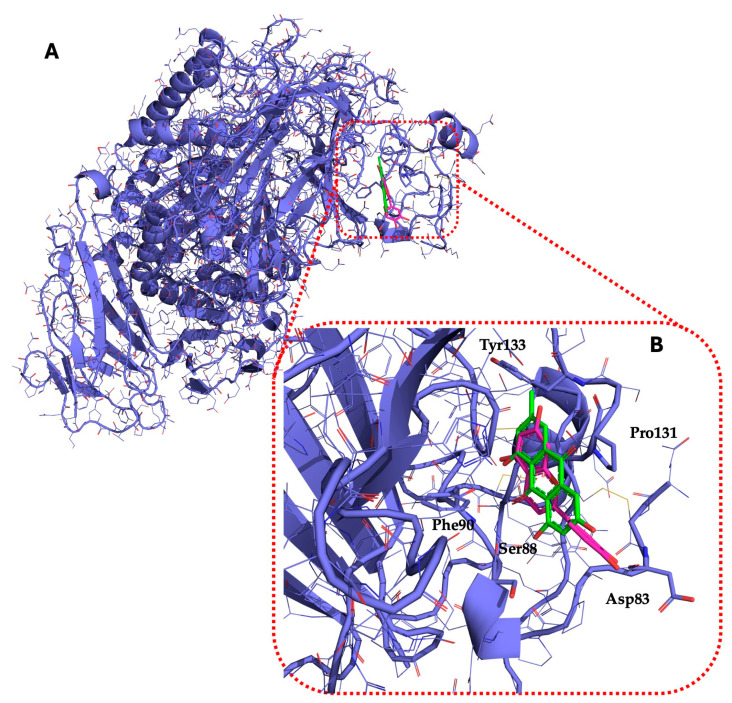
*(***A**) Representation of luteolin (magenta) and emodin (green) in α-glucosidase (blue); (**B**) Luteolin (magenta) and emodin (green) show overlapping binding at the α-glucosidase binding site. The highlighted amino acids interact with two compounds.

Luteolin and emodin exhibit binding in the same pocket (see Figure 4B); luteolin forms hydrogen bonds with Pro131, Asp83, Phe90, and Ser88, whereas emodin interacts with Phe90 and Ser88 (Figure 4B). Additionally, three other compounds—aloesin, lututeolin 6-c glucoside, and aloinoside B—also bind to the same pocket as luteolin and emodin, while no compound binds in the acarbose binding site.

## 3. Discussion

The findings from this study demonstrate the significant potential of *A. microcarpus* extracts in inhibiting the enzyme α-glucosidase. The EE exhibited strong inhibitory effects on α-glucosidase while showing minimal inhibition of α-amylase. This selective inhibition is beneficial as it aligns with the therapeutic goal of managing postprandial hyperglycemia in T2D without the gastrointestinal side effects associated with excessive α-amylase inhibition.

The inhibitory activity against α-glucosidase observed in the EE indicates a promising avenue for further development of *A. microcarpus* as a natural antidiabetic agent. The IC_50_ values for the EE are significantly lower than those of acarbose, a commonly used antidiabetic drug, suggesting that *A. microcarpus* extracts are highly potent inhibitors of α-glucosidase.

Additionally, the cell viability assays using Caco-2 cells confirmed that the EEs are not cytotoxic at concentrations up to 10 µg/mL, with cell viability remaining above 84%. The antioxidant activity and antimycotic assays further support the therapeutic potential of these extracts. The significant reduction in H_2_O_2_-induced ROS levels in Caco-2 cells treated with *A. microcarpus* extracts highlights their ability to mitigate oxidative stress, a common complication in diabetes.

The presence of phenolic compounds, particularly luteolin and its derivatives, is likely responsible for the observed biological activities [24]. Previous studies have shown that luteolin has strong inhibitory effects on both α-glucosidase and α-amylase, as well as potent antioxidant properties. The high concentrations of these compounds in the EE of *A. microcarpus* correlate well with the observed enzyme inhibition and antioxidant effects. Subsequently, we aimed to identify the specific compounds within the EE responsible for the inhibitory activity against α-glucosidase. Using molecular docking, we analyzed the interactions of 11 carefully selected compounds. These compounds included aloesin, which is present in all four ethanol extracts (S, L, T, and F), along with four compounds found in extract T and six compounds common to extracts S, L, and F. This selection ensured a comprehensive evaluation of potential inhibitors. The docking results revealed that only emodin, present in the T extract, and luteolin, present in three extracts (S, L, and F), exhibited significant binding affinity with α-glucosidase, suggesting their potential as inhibitors. The consistent presence of aloesin across all extracts had initially suggested its possible key role in the inhibition mechanism. However, this was not confirmed by the molecular docking studies and in vitro experiments.

In conclusion, this study underscores the therapeutic potential of *A. microcarpus* extracts in the management of T2D. The strong inhibitory effects on α-glucosidase, coupled with the antioxidant properties and lack of cytotoxicity, make these extracts promising candidates for further development as natural antidiabetic agents. On the other hand, these formulations have also demonstrated the ability to hinder the development of pathogenic *C. albicans* biofilm. This outcome is intriguing to contemplate, given that Candida infection frequently qualifies as a “biofilm-related infection”. It is a condition that is particularly difficult to treat because it frequently escapes antimitotic treatments and it is especially common in diabetic patients.

Future research should focus on the isolation and characterization of individual bioactive compounds, their pharmacokinetics, and in vivo efficacy to fully elucidate the therapeutic potential of *A. microcarpus*.

## 4. Materials and Methods

### 4.1. Plants Materials

Samples of *A. microcarpus* subsp. *microcarpus* Salzm. et Viv., including stems, leaves, flowers, and tubers (S, L, F, and T), were collected. After identification, a voucher specimen (number 1405/16) was archived in the Herbarium of the Life and Environmental Sciences Department, CAG. Following collection, the plant materials were thoroughly cleansed with deionized water. To preserve their integrity and constituents, the samples were immediately frozen at a temperature of −80 °C. Subsequently, they were lyophilized in their original form to ensure the preservation of their natural structure and composition. The lyophilized plant material was pounded, and one gram was used for each extraction process. The materials were extracted using ethanol (ethanol extract, EE) for 24 h at room temperature, ensuring constant stirring to maximize efficiency. The extracts were then filtered and lyophilized to obtain dry powders. Before use, 1 mg of dried powders was dissolved in DMSO (1 mL) for EE.

### 4.2. α-Amylase Inhibitory Activity

α-Amylase inhibitory activity of extracts was evaluated using 2-chloro-4-nitrophenyl-α-D-maltotrioside (CNPG3) as a synthetic substrate. The assay mixture comprised 60 µL of 50 mM sodium phosphate buffer (pH 7.0), 20 µL of 1 M NaCl, and 40 µL of α-amylase from porcine pancreas (concentration 1 mg/mL). The mixture was incubated at 37 °C for 10 min in the absence or presence of extract. Acarbose was used as a standard inhibitor for comparison. After incubation, 80 µL of CNPG3 solution (2.5 mM) was added. The release of 2-chloro-nitrophenol, due to enzymatic hydrolysis, was monitored spectrophotometrically at 405 nm [25].

### 4.3. α-Glucosidase Inhibitory Activity

The procedure to assess the inhibitory activity of α-glucosidase consisted of 120 µL 0.1 M phosphate buffer (pH 6.8) and 40 µL of *Saccharomyces cerevisiae* enzyme solution (0.125 U/mL) extract (20 µL) at various concentrations mixed and incubated for 15 min at 37 °C. Then, 20 µL of the substrate p-nitrophenyl α-D-glucopyranoside (pNPG), at a concentration of 5 mM in 0.1 M phosphate buffer, was added and the mixture was incubated again under the same conditions. The reaction was halted by adding 50 µL of 0.2 M sodium carbonate. The *p*-nitrophenol produced in the reaction was quantified at 405 nm using a 96-well microplate reader. DMSO was used as a control, ensuring its final concentration stayed below 8% *v*/*v* to avoid any impact on enzyme activity. Acarbose again served as a positive control. The IC_50_ value, indicating the concentration needed to inhibit 50% of α-glucosidase activity, was determined under the assay conditions.

### 4.4. Antimycotic Activity

A multidrug-resistant (MDR) isolate of *C. albicans* (CA97) was used for the antifungal susceptibility test by using the microplate dilution method. On this strain we have evaluated the minimum inhibitory concentration (MIC) and minimum biofilm inhibitory concentration (MBIC) of *A. microcarpus* extracts.

The MIC protocol was performed following the Clinical and Laboratory Standards Institute (CLSI) by using the broth microdilution procedure [26,27]. Briefly: the experiment was performed in sterile 96-well microplates (Corning, CLS3788 Merck KGaA, Darmstadt, Germany) containing (1:2) serial dilutions of *A. microcarpus* extract (200 μL) diluted in Sabouraud dextrose broth (BD, Maryland, USA), and the final concentration ranged from 4 to 1.9 × 10^−^^3^ mg/mL. Therefore, we added to each well a suspension of *C. albicans* until we reached a final concentration of 10^5^ CFU/mL in each well. The microplate was incubated at 37 °C in air. After 48 h of incubation, the plates were read with a microplate reader at 620 nm (Multiskan FC, Thermo Scientific, Monza, Italy). The MIC was the lowest concentration of an antimicrobial that showed the same turbidity value of the negative control (i.e., *Candida* suspension without formulate).

The biofilm protocol: We performed the evaluation of MBIC according to the modified crystal violet staining protocol, as previously reported [27]. *C. albicans* inoculum and the dilution formulation procedures were the same as those already described for the MIC experiments [27]. After 48 h of incubation time, the wells were gently washed three times with 150 μL of 0.9% saline solution. We then left the plate to dry for 30 min at 20 °C in a sterile laminar flow cabinet. Next, 200 μL of 20% acetic acid solution (Sigma) was added to each well, and biofilm was detached by vigorous pipetting. We conducted the experiment using three replicates and six wells serving as the negative control, containing only the growth medium. The plates were read with a microplate reader at 620 nm (Multiskan FC, Thermo Scientific) at 450 nm. The MBIC represented the lowest concentration of formula able to interfere with biofilm formation; in other words, the dilution showed the same absorbance value as the negative control.

### 4.5. Cell Viability

The MTT assay technique on Caco-2 cells was employed to assess cell viability. Caco-2 cells were maintained in standard conditions, with 5% CO_2_, 95% relative humidity, and a temperature of 37 °C. The culture medium used was Dulbecco’s modified Eagle’s medium, enhanced with 1% penicillin/streptomycin (Euroclone, Milan, Italy) and supplemented with 10% fetal bovine serum (Gibco, Grand Island, NY, USA).

The cells were plated in 96-well plates at a density of 5 × 10^3^ cells per well, treated with extracts at different concentrations (0–10 µg/mL) and then incubated for 24 h. Subsequently, an MTT solution (0.5 mg/mL) was added to cells and incubated for 3 h at 37 °C. The formazan crystals formed were dissolved in DMSO and the absorbance was measured at 590 nm to assess cell viability.

### 4.6. Dichlorofluorescein (DCF) Assay (or Intracellular ROS Levels)

The 2′,7′-dichlorofluorescein diacetate (DCFH-DA) method was used to measure intracellular reactive oxygen species (ROS) levels and evaluate the antioxidant activity of the extracts. Caco-2 cells were treated with the extract at various concentrations (0–10 µg/mL) for 24 h. Following this, the cells were incubated with 10 µM DCFH-DA at 37 °C for 30 min. After incubation, 2 mM H_2_O_2_ was added to each well. The fluorescence intensity of DCF was then measured at the excitation wavelength of 485 nm and emission wavelength of 530 nm. The fluorescence data were recorded over 60 min to evaluate the antioxidant activity of the samples.

### 4.7. (HR) HPLC-ESI-QToF MS and (HR) HPLC-ESI-QToF MS/MS Analysis of A. microcarpus Extracts

For the qualitative assessment of the plant extracts, the method outlined by Di Petrillo et al. [13] with some modifications [28] was used. Briefly, the analytical setup included an advanced ion mobility QToF LC/MS system equipped with a 1290 Infinity II UPLC and a 6560 IM-QToF (Agilent Technologies Inc., Palo Alto, CA, USA) and experiments were conducted using an electrospray ionization (ESI) source, set to operate in negative ion mode. Data acquisition and processing were performed using the Agilent MassHunter Workstation Acquisition software v. B.09.00 (Agilent Technologies, Palo Alto, CA, USA). ESI/QToF MS data were then analyzed using the MassHunter Workstation Qualitative Analysis software v. 10.0 (Agilent Technologies) and the MassHunter METLIN metabolite PCDLdatabase v. B.08.00 (Agilent Technologies) and Sirius^®^ software v. 4.7.4 were used for the tentative identification of the metabolites and predict fragmentation and molecular formulae [22,29], along with comparing experimental MS/MS spectra with fragmentation patterns reported in the literature or with spectra reported in a public repository of mass spectral data (KNApSAcK Core System, n.d.).

### 4.8. Statistical Analyses

Statistical differences were evaluated using GraphPad software Version 8 (GraphPad Software, San Diego, CA, USA). Comparison between groups was assessed by one-way ANOVA followed by the Tukey test. The values with *p* < 0.05 were considered significant.

### 4.9. In Silico Studies

#### 4.9.1. Protein and Ligand Preparation

The crystal structure of α-glucosidase 5NN4 [30] was selected from Protein Data Bank (RCSB PDB) [31]. Co-crystallized ligands were removed, and the resulting structure was prepared using the Protein Preparation Wizard (Schrödinger Release 2022-3), all hydrogen atoms were added, bond orders were adjusted, and formal charges were assigned. The appropriate ionization state was determined at pH 7.4 using the PROPKA tool. The protein minimization by OPLS4 force field to fix all molecular overlaps and strains was then performed. The restrained minimization was terminated when the average root mean square deviation (RMSD) of the non-hydrogen atoms was converged to 0.3 Å.

The compounds for docking were selected based on the results of compound identification in *A. microcarpus*. Specifically, aloesin, present in all extracts, eight compounds present in S, L, and F extracts, and emodin found solely in the T extract were chosen for further analysis. All ten compounds were subjected to conformational analysis using Quantum Mechanic (QM) Conformer and Tautomer Prediction by Jaguar (Schrödinger Release 2022-3) [32]. The best conformations obtained were prepared using LigPrep (Schrödinger Release 2022-3) preserving the specified chiralities, employing the OPLS4 force field.

#### 4.9.2. Molecular Docking

Gasteiger charges [33] were assigned and the grids were generated using AutoGrid4.2 using whole protein. For AutoDock4.2 [34], docking experiments were carried out using the Lamarckian genetic algorithm [35,36], a total of 250 runs were performed with a population size of 250 individuals, and maximum numbers of evaluations were set at 2,500,000. Other parameters were kept as default.

## 5. Conclusions

This study highlights the promising potential of *Asphodelus microcarpus* extracts as effective natural inhibitors of α-glucosidase, a critical enzyme in the management of T2D. The findings demonstrate that the ethanolic extracts of *A. microcarpus* exhibit strong inhibitory activity on α-glucosidase, with IC_50_ values lower than that of acarbose, the standard antidiabetic drug. Conversely, the extracts showed minimal inhibition of α-amylase, indicating a beneficial selectivity that could help mitigate the gastrointestinal side effects commonly associated with excessive α-amylase inhibition.

Additionally, the extracts displayed antioxidant properties and were found not to be cytotoxic. This suggests that *A. microcarpus* not only holds potential for glycemic control but also may contribute to the reduction of oxidative stress, a significant factor in the complications of diabetes.

Further molecular docking studies identified luteolin and emodin as significant binders to the α-glucosidase enzyme, hinting at their roles as key bioactive components contributing to the observed enzyme inhibition. Although aloesin, present in all extracts, initially appeared relevant, it did not demonstrate the same binding affinity, indicating that the therapeutic potential lies more strongly with luteolin and emodin. Furthermore, these formulations have proven to effectively inhibit the growth of pathogenic *C. albicans* biofilm.

In summary, *A. microcarpus* represents a valuable source of natural compounds with potential application in T2D management, offering safer and effective alternatives or adjuncts to conventional treatments.

## Figures and Tables

**Table 1:** Inhibition (%) of *A. microcarpus* ethanolic extracts (EEs) at 100 μg/mL against the enzymes α-glucosidase and α-amylase.

Extract	α-Glucosidase	α-Amylase
Stems	97.6 ± 2.8	13.5 ± 0.3
Leaves	88.8 ± 5.1	20.6 ± 0.6
Flowers	92.3 ± 4.2	14.6 ± 0.4
Tubers	98.2 ± 3.7	20.2 ± 0.9

**Table 2:** IC_50_ value of *A. microcarpus* EE against α-glucosidase.

Extract	IC_50_ (µg/mL)
Stems	4.3 ± 0.5
Leaves	3.8 ± 0.4
Flowers	4.6 ± 0.4
Tubers	3.6 ± 0.1
Acarbose	90 ± 7.3

**Table 3:** Antibiofilm pattern of *A. microcarpus* EE against an MDR strain of *C. albicans*.

Extract	MIC (mg/mL)	MBIC (mg/mL)
Stems	>4 mg/mL	1.3 mg/mL
Leaves	>4 mg/mL	1.3 mg/mL
Flowers	>4 mg/mL	1.3 mg/mL
Tubers	>4 mg/mL	4 mg/mL

**Table 4:** Compound identification by (HR) HPLC-ESI-QToF MS/MS in *A. microcarpus* extracts (X, present; -, absent).

Peak N°	R_t_	[M−H]^−^ *m*/*z*	Molecular Formula	Δ mDA	MS/MS *m*/*z* (%)	Tentative Identification	S E E	L E E	T E E	F E E	References
1	9.29	393.1203	C_19_H_22_O_9_	1.225	273.0774 (100)/ 245.0807 (52)	Aloesin	X	X	X	X	[18,21]
2	9.69	353.0888	C_16_H_17_O_9_	1.68	179.0341 (27)/ 191.0561 (67)	3-O-caffeoylquinic acid	X	X	-	X	[13,15]
3	10.77	351.1307	C_14_H_24_O_10_	1.77	101.0598 (100)	Alkyl glycoside	X	X	-	-	[22]
4	10.81	269.0469	C_15_H_10_O_5_	−0.98	225.0571 (100)	Emodin	-	-	X	-	[19]
5	11.23	353.0888	C_16_H_17_O_9_	1.68	179.0352 (33)/ 191.0561 (85)	5-O-caffeoylquinic acid	X	X	-	X	[13,15]
6	11.78	509.1684	C_24_H_30_O_12_	0.88	257.0827 (100)	Unknown	-	-	X	-	[22]
7	12.36	337.0938	C_16_H_18_O_8_	1.22	191.0560 (77)	Coumaroyl quinic acid	X	X	-	-	[17]
8	13.04	335.0778	C_16_H_16_O_8_	1.43	179.0341 (86)/ 135.0447 (100)	5-O-Caffeoilshikimic acid	X	-	-	-	[21]
9	14.41	399.1668	C_16_H_18_O_8_	1.81	175.1115 (100)	Alkyl glycoside	X	X	-	-	[22]
10	15.49	447.0948	C_21_H_20_O_11_	1.70	357.0626 (100)/ 327.0515 (88)	Luteolin 6-C-glucoside	X	X	-	X	[13,15]
11	18.20	461.1107	C_22_H_22_O_11_	1.56	254.0560 (100)	Unknown	X	X	-	-	
12	19.06	475.0899	C_22_H_20_O_12_	1.65	431.0982 (100)	Unknown	X	X	-	-	
13	19.25	489.1054	C_23_H_22_O_12_	1.38	429.0790 (25)/ 327.0562 (100)	Luteolin acetyl glucoside	X	X	-	X	[13,15]
14	20.51	539.1794	C_25_H_32_O_13_	0.01	215.0714 (100)	Unknown	-	-	X	-	[22]
15	22.70	577.1577	C_27_H_30_O_14_	0.01	253.0507 (100)	Chrysophanol diglucoside	-	-	X	-	[22]
16	23.93	563.1778	C_27_H_32_O_13_	0.01	239.0712	Aloinoside A/B	-	-	X	-	[20]
17	26.06	949.2811	C_40_H_54_O_26_	−3.87	859.2484 (100)	Unknown	-	-	X	-	[22]
18	26.15	285.0406	C_15_H_10_O_6_	0.10	-	Luteolin	X	X	-	X	[13,15]
19	29.66	313.0358	C_16_H_10_O_7_	1.46	269.0456 (100)	Laccaic acid	X	-	-	-	[21]
20	30.07	933.2841	C_47_H_50_O_20_	−3.38	843.2480 (100)	Unknown	-	-	X	-	[22]
21	30.81	961.2789	C_41_H_54_O_26_	4.01	533.1256 (75)/ 490.1066 (100)	Unknown	-	-	X	-	[22]
22	31.91	327.2189	C_18_H_32_O_3_	−1.08	239.1312 (56)	Unknown	X	X	X	-	[21]
23	32.65	933.2820	C_47_H_50_O_20_	−1.22	843.2517 (100)	Unknown	-	-	X	-	[22]
24	32.86	242.1763	C_13_H_25_NO_3_	0.15	225.1512 (100)/181.1594 (88)	Amino-oxotridecanoic acid	X	X	X	-	[22]

**Table 5:** Graphical representation and results of docked compounds and their provenance in *A. microcarpus*.

Compounds	Name	Binding Affinity Kcal/mol	Identified in *A. microcarpus* Extracts
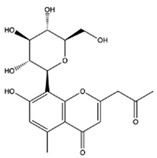	Aloesin	−4.70	ALL
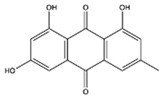	Emodin	−6.14	TEE
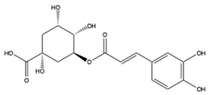	3-*O*-caffeoylquinic acid	−4.87	SEE; LEE; FEE
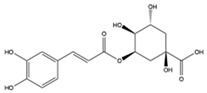	5-*O*-caffeoylquinic acid	−4.15	SEE; LEE; FEE
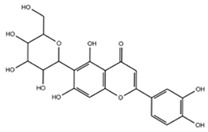	Luteolin 6-*C*-glucoside	−3.82	SEE; LEE; FEE
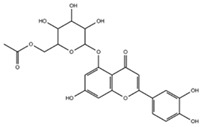	Luteolin 5-*O* acetyl glucoside	−4.11	SEE; LEE; FEE
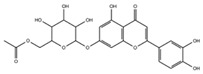	Luteolin 7-*O* acetyl glucoside	−4.63	SEE; LEE; FEE
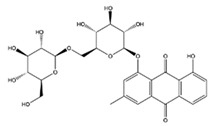	Chrysophanol diglucoside	−2.09	TEE
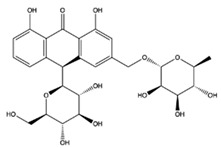	Aloinoside A	−4.57	TEE
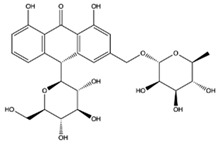	Aloinoside B	−3.43	TEE
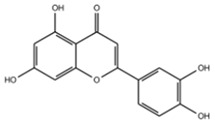	Luteolin	−6.65	SEE; LEE; FEE

## Data Availability

The data underlying this article are available in the article.
